# Shear Mode Bulk Acoustic Resonator Based on Inclined *c*-Axis AlN Film for Monitoring of Human Hemostatic Parameters

**DOI:** 10.3390/mi9100501

**Published:** 2018-09-30

**Authors:** Shuren Song, Da Chen, Hongfei Wang, Chaohui Li, Wei Wang, Wangli Yu, Yanyan Wang, Qiuquan Guo

**Affiliations:** 1 College of Electronics, Communications, and Physics, Shandong University of Science and Technology, Qingdao 266590, China; sdustsongshuren@sina.com (S.S.); phywjj@163.com (H.W.); eechaohui_li@163.com (C.L.); skdwangwei1zqf@163.com (W.W.); 2 State Key Laboratory of Mining Disaster Prevention and Control Co-Founded by Shandong Province and the Ministry of Science and Technology, Shandong University of Science and Technology, Qingdao 266590, China; 3 Zhuhai Topsun Electronic Technology Co., Ltd., Zhuhai 519060, China; pswlyu@163.com; 4 School of Optoelectronic Science and Engineering & Collaborative Innovation Center of Suzhou Nano Science and Technology, Soochow University, Suzhou 215006, China; yywang@suda.edu.cn; 5 Mechanical & Materials Engineering, University of Western Ontario, London, ON N6A 3K7, Canada

**Keywords:** bulk acoustic wave, AlN film, hemostatic parameters, viscosity sensor

## Abstract

Measurement of hemostatic parameters is essential for patients receiving long-term oral anticoagulant agents. In this paper, we present a shear mode bulk acoustic resonator based on an inclined *c*-axis aluminum nitride (AlN) film for monitoring the human hemostatic parameters. During the blood coagulation process, the resonant frequency of the device decreases along with a step-ladder profile due to the viscosity change during the formation of fibers in blood, revealing the sequential coagulation stages. Two hemostatic parameters with clinical significance, prothrombin time (PT) along with its derived measure of international normalized ratio (INR), are determined from time-frequency curves of the device. Furthermore, the resonator is compared with a commercial coagulometer by monitoring the hemostatic parameters for one month in a patient taking the oral anticoagulant. The results are consistent. In addition, thanks to the excellent potential for integration, miniaturization and the availability of direct digital signals, the proposed device has promising application for point of care coagulation monitoring.

## 1. Introduction

Oral anticoagulants are very commonly used for the management of chronic conditions such as angiocardiopathy, thrombosis, pulmonary embolism and the potential sequelae of valve replacement [1]. In these therapeutic situations, the dosage of anticoagulants must be carefully controlled to keep a sufficient level of anticoagulation while not increasing the risk of excessive bleeding. In fact, many factors in daily life including diet, exercise, drinking, infection and concomitant drug therapy may influence the efficacy of oral anticoagulants [2]. This makes it necessary to closely monitor the hemostatic parameters, such as prothrombin time (PT), activated partial thromboplastin time (APTT), thrombin time (TT) and international normalized ratio (INR) [3], during the treatment. In the hospital, the hemostatic parameters are usually measured by thromboelastography (TEG), paramagnetic particle methods or optical methods based on the change in viscoelasticity or turbidity during the blood coagulation process [4,5]. However, the existing testing methods require milliliter-scale sample, macroscale set-up and trained operators, and thus are not suitable for point of care (POC) use. As a result, there is still a particular desire for sensors that are miniaturized, low-cost, simple in operation and allow self-testing at home.

Bulk acoustic resonators, represented by quartz crystal microbalance (QCM), appear to be ideal for a POC system thanks to their simple structure and the absence of macroscopically moving parts [6]. The resonant states of QCM are very sensitive to the changes of liquid viscosity [7] and mass loading [8] on the resonator surface during the biological reactions. This feature allows QCM to monitor the blood coagulation process in real-time [9,10,11,12]. However, the typical QCM has the size of about 1 cm^2^, the frequency of 5–20 MHz and the gram-levels base mass because of the bulk structure, which results in a large detection limit, thick substrate and the difficulty to integrate with the electronics. During the last few years, film bulk acoustic resonator (FBAR), a promising micro-electromechanical bulk acoustic resonator, is popular for low-cost, label-free and highly sensitive detection of gas [13,14,15,16] and biochemical species [17,18,19,20]. By using micron-thick piezoelectric ZnO or AlN thin film, FBAR brings a very high resonant frequency from sub-GHz to 10 GHz and a low detection limit down to the monomolecular level.

Most FBAR has a similar structure to that of QCM and its working mechanism is also based on the same principle. Usually, the piezoelectric thin films are deposited with normal-plane *c*-axis crystal orientation and sandwiched between two electrodes, which makes FBAR operate in thickness longitudinal mode (TLM) [21,22,23]. However, when these devices work in viscous liquids such as blood, tissue fluids and biocolloids, the longitudinal wave is radiated into the liquid through compressional motion, resulting in a serious damping [24,25,26]. Conversely, the thickness shear mode (TSM) wave does not compress and hardly propagates in liquids [27,28]. Therefore, the TSM FBAR maintains high Q factor and sensitivity to the tiny change in mass or viscosity on the solid-liquid interface in liquid environment. Although the TSM FBAR devices have been demonstrated for the detection of anti-avidin [29], DNA [30], narcotics [31], and organophosphorus [32], the studies on TSM FBAR sensors are still limited in terms of the biosensing applications compared with the widely reported TLM FBAR and QCM devices. In addition, Xu et al. [33] proposed a special contour-mode FBAR device with a suspended ring of piezoelectric film for monitoring the blood coagulation. So far, the FBAR devices were not applied to analyze the coagulation disorders of real patients’ samples.

It is proved that TSM mode resonances can be excited when there is a component of the field perpendicular to the *c*-axis in the wurtzite piezoelectric polycrystals [34]. An easy exciting approach is to employ lateral electric field through coplanar electrodes on the surface of piezoelectric films with normal-plane *c*-axis orientation. Based on this method, the lateral excited FBAR devices show significantly improved performances in viscous media. We have reported the lateral excited FBAR as a viscosity sensor for the blood coagulation measurements [35,36,37]. However, an obvious shortcoming of this method is the relatively weak electric field between the electrodes with the gap of several microns, resulting in a very low electromechanical coupling [38] (see Supplementary Material). More importantly, the liquid is in direct contact with both of the electrodes and hence deteriorates substantially the sensor performances. In order to alleviate the deficiency of lateral excitation and enhance the practicality for biosensing, some efforts have been made to grow inclined *c*-axis-oriented crystal columns coupled with two electrodes situated on the opposite sides of the piezoelectric films [24,39,40]. In this situation, the shear mode resonance is excited by the electric field along thickness direction. The sandwiched structure allows the elimination of electrical disturbance from the liquid, which is very beneficial for acquiring reliable results for the clinic.

In this work, we describe a TSM FBAR biosensor based on inclined *c*-axis AlN films for coagulation analysis. The electro-acoustic responses were characterized when the device operated in air, water, blood and viscous liquids. Then the resonant frequency was correlated to the viscosity change during the blood coagulation process. Two hemostatic parameters with the clinical significance, PT and its derived measure of INR, were measured by the FBAR biosensor as a demonstration. The FBAR biosensor permits simple determination of the hemostatic parameters of patients in a fast manner. The results were consistent with the traditional coagulometric method used in clinical practice. As far as we know, this is the first report on the use of FBAR biosensors in a patient’s long-term coagulation monitoring.

## 2. Experimental Studies

### 2.1. Device Structure

**Figure 1:**
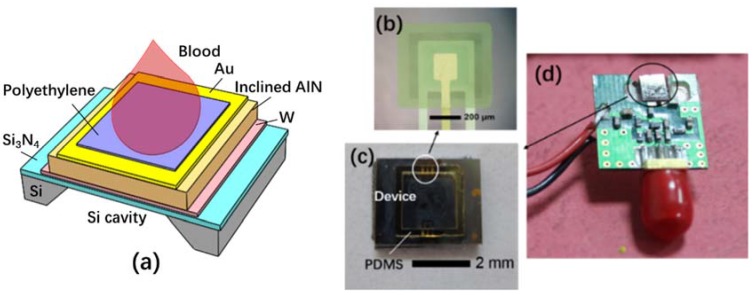
Film bulk acoustic resonator (FBAR) biosensor based on inclined *c*-axis aluminum nitride (AlN) film for the hemostatic tests: (**a**) the schematic illustration of the FBAR biosensor; (**b**) the micrograph of the fabricated device; (**c**) the piece including the test pool and the FBAR sensor; (**d**) the picture of the printed circuit board (PCB) assembled with FBAR biosensor.

Figure 1a shows the schematic illustration and the photograph of the FBAR biosensor. The piezoelectric AlN film (1.5 μm) is sandwiched with W (100 nm) and Au/Ti electrodes (100 nm/10 nm) on a 0.6 μm-thick Si_3_N_4_ membrane. The substrate under the piezoelectric stack is removed to form an air-solid interface for the resonance. A testing pool with a dimension of 2 mm × 2 mm × 1 mm was fabricated with the sidewall of Polydimethylsiloxane (PDMS) to make the resonator surface have a good contact with the blood samples. A thin hydrophilic polyethylene layer was coated on the resonator surface for the convenience of blood contact and fibrin buildup during the coagulation process. A Colpitts oscillator circuit was assembled to monitor the resonant frequency using a network analyzer (Agilent 8714 ET, Agilent, Santa Clara, CA, USA).

### 2.2. Device Fabrication

**Figure 2:**
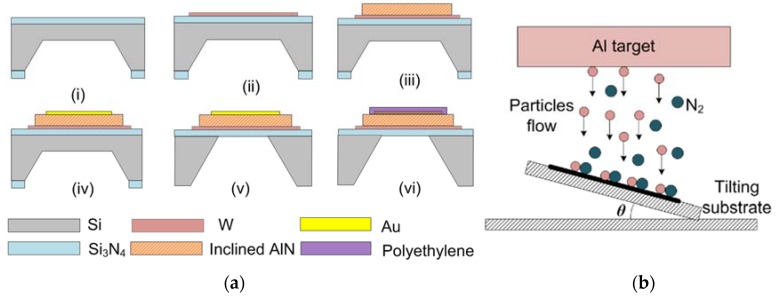
(**a**) Fabrication process of the FBAR: (**i**) Etching of one side of the Si wafer with the mask of Si_3_N_4_; (**ii**) Deposition and pattern of bottom W electrode; (**iii**) Deposition and pattern of the AlN film with inclined *c*-axis; (**iv**) Preparation of top Au/Ti electrode; (**v**) Dry etching of the residual silicon; (**vi**) Spin-coating of polyethylene layer. (**b**) Sketch of the tilting substrate for AlN deposition (*θ* = 30°).

The fabrication process of the FBAR is shown in Figure 2. The process started with a (100) Si wafer with low-stress Si_3_N_4_ layers. First, one side of the wafer was wet etched by KOH at 80 °C to form an initial cavity. Then, the AlN film and electrodes were sputtered and patterned on the other side of the wafer using a radio frequency (RF)/DC magnetron sputtering system (JSD400, ZKY Co., LTD., Shenyang, China). At last, the residual Si was removed by deep reactive ion etching (601E, Alcatel, Paris, France) to isolate the resonator acoustically from the substrate. The polyethylene solution in Decalin (0.5%, Aladdin, Shanghai, China) were spin-coated on the top electrode of the device at a rotation speed of 6000 rpm for 40 s and then annealed at 60 °C for 20 min.

In order to grow the AlN film with inclined *c*-axis, a tilting substrate holder (*θ* = 30°) was used as shown in Figure 2b. The particles flux from the target is angled with respect to the substrate surface. The sputtering process was performed at a pressure of 0.5 Pa with a 40% N_2_ in Ar gas admixture and an RF power of 1200 W. The AlN film was examined by X-ray Diffraction (XRD, D/Max 2500, Tokyo, Rigaka, Japan) and field emission scanning electron microscope (FE-SEM, S-4800 Hitachi, Tokyo, Japan).

### 2.3. Measurement Procedure

The blood samples were collected from five healthy donors and a patient taking oral anticoagulants (warfarin, 4 mg daily, Orion Corporation, Espoo, Finland) using the medical blue vacutainers (3.2% sodium citrate, 1:9). The collected samples were stored at room temperature (20 °C) after blood draw and the coagulation experiments started within 20 min. For the measurement of PT, the fresh blood samples were pretreated following the specifications recommend by the vendor of commercial coagulometer (Urit Medical Equipment Co., Ltd., Guilin, China). First, one microliter PT test reagent (including thromboplastin reagent and CaCl_2_, supplied by Urit Medical Equipment Co., Ltd., Guilin, China) was dropped onto the FBAR surface using a pipette. After a few seconds, equal volume of citrated blood was dropped to trigger the coagulation. The change of blood viscosity was monitored by following the frequency response in real time.

In order to verify the reproducibility of the FBAR biosensor, the blood samples were further diluted with Tris buffer (pH 7.4) (Hepalink Pharmaceutical Co., Ltd., Shenzhen, China) in different ratio. The same samples were also tested by a commercial mechanical coagulometer (URIT-600, Guilin Urit Medical Equipment Co., Ltd., Guilin, China) to compare the results. All the measurements were performed at room temperature.

According to the definition of clinical diagnosis, INR is a normalized parameter that adjusts for changes in the PT reagents and allows for results from different laboratories and test methods to be comparable. International normalized ratio is determined from the measured PT and the international sensitivity index (ISI) of the testing reagent as follows:

(1) $$INR={\left(\frac{PT_{test}}{PT_{normal}}\right)}^{ISI}$$

where *PT_test_* is the prothrombin time for the blood sample, and *PT_normal_* is the mean prothrombin time from healthy groups (1.0–1.2) measured by the identical instrument. The ISI is provided by the vendor of the testing reagent and indicates how a batch of tissue factor compares to an international reference tissue factor.

## 3. Results and Discussion

### 3.1. Characterization of the AlN Film with Inclined c-Axis

**Figure 3:**
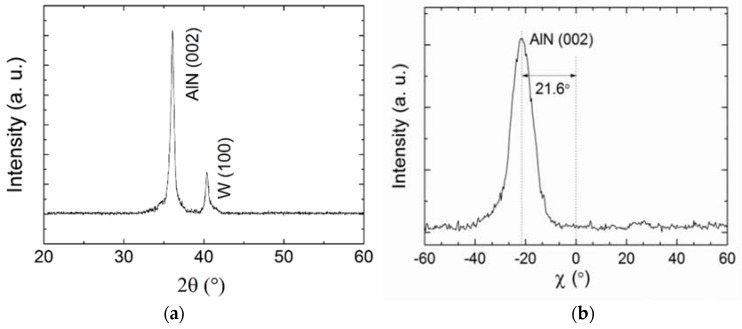
X-ray diffraction (XRD) patterns of the AlN film. (**a**) 2*θ* scan; (**b**) *χ* scan of the AlN (002) peak, revealing a 22° inclination with a full width at half-maximum (FWHM) of 10.2°.

**Figure 4:**
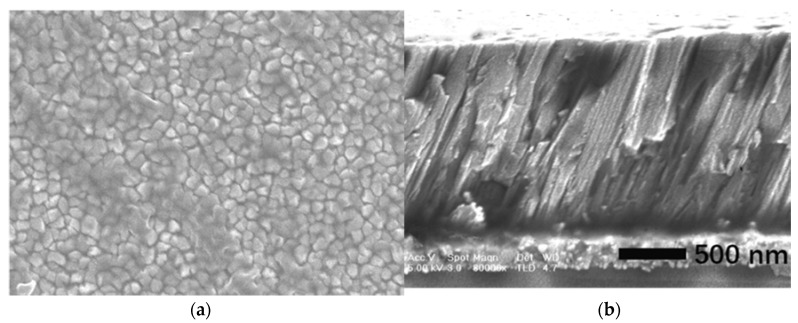
Scanning electron microscopy (SEM) pictures of the AlN film. (**a**) Surface morphology; (**b**) Cross-section view.

Figure 3 shows the XRD patterns of the AlN film. There is a (002) AlN preferential orientation (2*θ* ≈ 36°) besides the small peak corresponding to W electrode (2*θ* ≈ 40°). The *χ* scan pattern shows that the *c*-axis of the AlN film is 21.6° inclined to the normal direction. The full width at half-maximum (FWHM) of *χ* peak of 10.2° suggests a broad angular spreading of the inclination angle in different grains. As shown in Figure 4, the dense-packed and inclined crystal columns are clear. For the further improvement of crystal quality and orientation uniformity, the two-step sputtering process could be employed to deposit a thin seed layer [38,41]. Another method was the introduction of blinds in the path between the target and the substrate [42].

### 3.2. Electro-Acoustic Response in Air, Water and Blood

The comparison of the exciting electric field, electromechanical coupling and noise level between the FBAR devices with sandwiched and coplanar electrodes were summarized in Table 1. The details of calculation and experimental results were shown in Figures S1–S4. Obviously, the sandwiched structure has the improved performances and thus is more suitable for biosensing applications.

**Figure 5:**
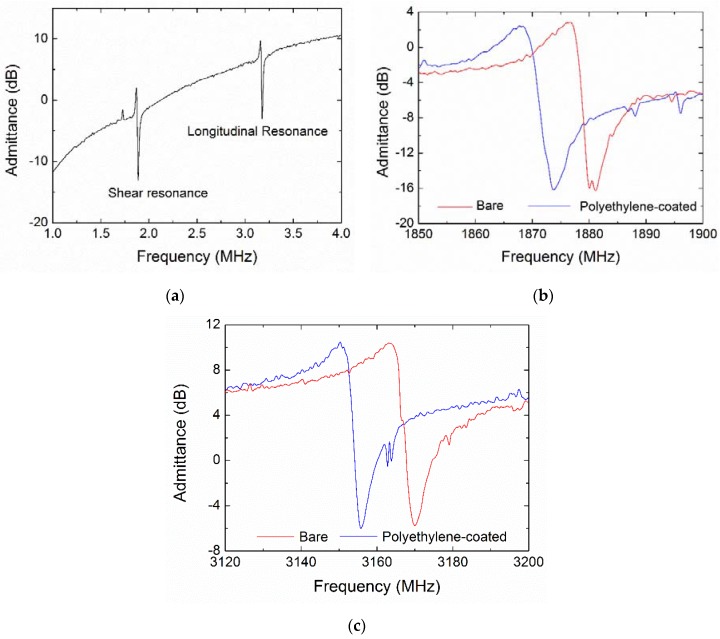
Admittance curves of the FBAR based on *c*-axis inclined AlN film measured in air. (**a**) The admittance curve of the bare device without polyethylene layer in a wide frequency range; (**b**) The detailed curves near the shear resonance before and after coating; (**c**) The detailed curves near the longitudinal resonance before and after coating.

Figure 5a shows the admittance curves of the bare FBAR device, illustrating the shear and longitudinal resonances to be near 1.87 GHz and 3.15 GHz, respectively. Generally, the resonant frequency (*f_R_*) of bulk acoustic devices can be estimated by *f_R_* = *v*/2*d*, where *v* and *d* are the acoustic velocity and thickness of the piezoelectric layer. From the resonant peaks in the conductance curves, the corresponding shear velocity *v*^(*S*)^ and longitudinal velocity *v*^(*L*)^ were 5610 m/s and 9450 m/s, respectively. As a comparison, the theoretical acoustic velocities of the AlN film with the *c*-axis inclination angle of 22° were calculated to be *v*^(*S*)^ = 6221 m/s and *v*^(*L*)^ = 10,268 m/s from the basic piezoelectric constitutive equations as follows [34]:

(2) $$v^{\left(S\right)}={\left[\frac{\bar{c_{33}^{E^{\prime }}}+\bar{c_{55}^{E^{\prime }}}}{2\rho }-\sqrt{{\left(\frac{\bar{c_{33}^{E^{\prime }}}-\bar{c_{55}^{E^{\prime }}}}{2\rho }\right)}^{2}+{\left(\frac{\bar{c_{35}^{E^{\prime }}}}{\rho }\right)}^{2}}\right]}^{1/2}$$

(3) $$v^{\left(L\right)}={\left[\frac{\bar{c_{33}^{E^{\prime }}}+\bar{c_{55}^{E^{\prime }}}}{2\rho }+\sqrt{{\left(\frac{\bar{c_{33}^{E^{\prime }}}-\bar{c_{55}^{E^{\prime }}}}{2\rho }\right)}^{2}+{\left(\frac{\bar{c_{35}^{E^{\prime }}}}{\rho }\right)}^{2}}\right]}^{1/2}$$

where *c^E^*^′^_33_ and *c^E^*^′^_55_ are the piezoelectrically stiffened elastic constants in the material coordinate system with inclined *c*-axis and *ρ* is the density of AlN film.

The polyethylene [11] and pyrrolidone-divinylbenzene [43] were proposed as the contacting layer between the blood samples and QCM devices to improve the hydrophilia and speed up the fibrin buildup during the coagulation process [44]. It was found that the coagulation times measured by the QCM devices with these coatings were well consistent with the results obtained from commercially available coagulometers [11,43,45].

The admittance details before and after coating of polyethylene were shown in Figure 5b,c. Table 2 summarizes the resonant frequencies and Q factors of the shear and longitudinal modes. Due to the mass-loading effect, the frequency decreased by 8.1 MHz and 12.9 MHz for the shear and longitudinal resonances, respectively. It is noted that the coating did not cause meaningful influence on Q factors for all the FBAR devices, suggesting that the cured polyethylene layer can be considered as a rigid load in the acoustic propagation path. The Q factor is defined from the 3-dB bandwidth near the resonant peak using the following equations [46,47]:

(4) $$Q={\left|\frac{f}{BW_{3dB}}\right|}_{f=f_{R}}$$

**Figure 6:**
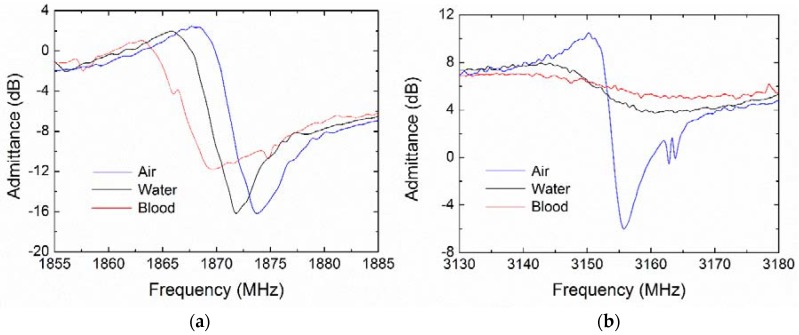
Admittance curves of the polyethylene coated FBAR based on *c*-axis inclined AlN film, measured in air, water and blood sample. (**a**) Near the shear resonance; (**b**) Near the longitudinal resonance.

When the FBAR works in a liquid environment, the compressional longitudinal wave propagates into the liquid, thus dissipating the wave energy. In contrast, the shear wave localizes its energy close to the resonator surface, causing most of the wave energy to be reflected into the piezoelectric stack with high efficiency. These features were evident for the FBAR biosensor working in air and blood as show in Figure 6. Compared with the case in air, the shear mode resonance exhibited only small frequency downshifts and slight degradation in water and citrated blood, whereas the longitudinal resonance was seriously deteriorated in water and even completely restrained in blood.

As outlined in Table 3, the shear mode Q factor decreases from 263 in air to 203 in water (by 22.8%) and 129 in blood (by 50.9%), respectively. However, the longitudinal mode Q factor is reduced by up to 88.1% in water, thus rendering the longitudinal resonance unsuitable for the biosensing applications in liquid environments.

### 3.3. Viscosity Characterization of Shear Resonance

**Figure 7:**
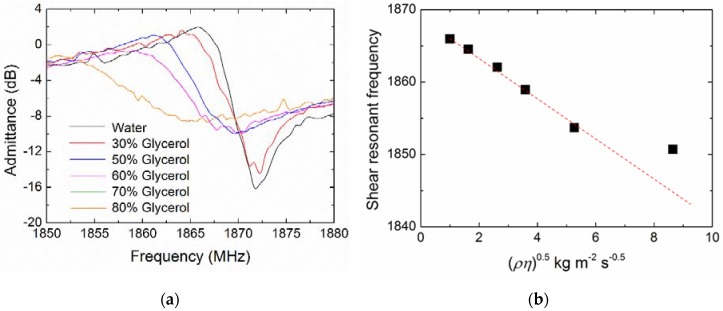

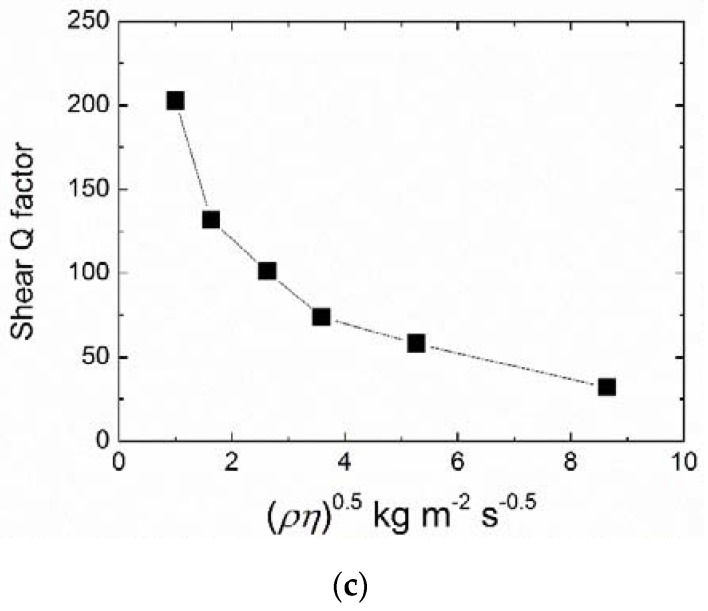
Viscous responses of the polyethylene-coated FBAR measured in water and glycerol solutions. (**a**) Admittance curves near the shear resonance; (**b**) Shear resonant frequency and (**c**) Q factor as a function of the square root of the product of the liquid density and viscosity (*ρη*)^0.5^.

Viscosity characterization of the FBAR biosensor was performed using standard aqueous glycerin solutions with concentrations from 0 to 80 wt.%, which corresponded to a viscosity of 1 to 0.0618 Pa·s. Figure 7a shows detailed admittance curves near the shear resonance measured in water and glycerin solutions. The damping effect caused a frequency downshift and an obvious broadening in the resonant peak with the increasing viscosity. Figure 7b,c show the resonant frequency and the Q factor as a function of the square root of the product of the liquid density and viscosity (*ρη*)^0.5^. In the low viscous liquids ((*ρη*)^0.5^ < 5.26 kg·m^−2^·s^−0.5^), the linear relation between the frequency shift and the value of (*ρη*)^0.5^ agreed well with the Newtonian fluid model predicted by the model of Kanazawa and Gordon [27] for the electroacoustic resonance. In the glycerol solutions with higher viscosity, the frequency shifts deviated from the linear dependence, suggesting a behavior of Maxwellian viscoelasticity [48].

### 3.4. Blood Coagulation Monitoring

**Figure 8:**
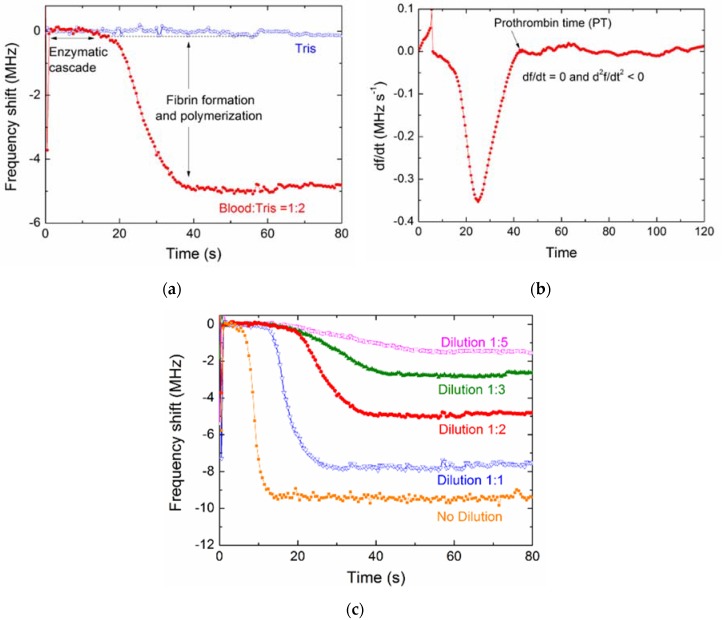
(**a**) Representative real-time frequency response of the polyethylene-coated FBAR for the coagulation process of diluted blood samples; (**b**) First derivative of the frequency-time curve; (**c**) Coagulation responses of the blood samples with different dilution (blood: Tris). All the blood samples were collected from the same health donor.

Figure 8 shows the representative real-time frequency responses of the polyethylene-coated FBAR for the coagulation process of diluted blood samples. A control measurement was performed using Tris buffer to exclude effects other than those due to coagulation, and its frequency response was given as the base line of the frequency shifts (*Δf*). There was an obvious difference between the curves of blood coagulation and the control. The frequency of the control without coagulation process leveled off to a plateau phase. However, the time-frequency curve showed a step-ladder profile during the blood coagulation process similar to that of other electroacoustic coagulation sensors [10,11,12].

Blood coagulation is a complex biological process involving the cascade enzyme reaction, fibrin formation and fibers polymerization [37], which can be revealed by following the frequency shift of FBAR in real time. First, the enzymatic cascade was triggered by calcium ions and thromboplastin. In this stage, the prothrombin was converted to thrombin by the catalytic action of prothrombinase complex and the blood viscosity had no obvious change [4,11], corresponding to the first plateau phase in the frequency profile as shown in Figure 8a. Subsequently, the enzymatic action of thrombin on the soluble protein fibrinogen present in blood generated the fibrin monomers that eventually polymerized into fibers. With the growth of fibers network on the FBAR surface, the blood gradually thickened and an additional mass was loaded on the solid-liquid interface, leading to a frequency decrease. After the formation of the gel-like clot, the viscosity and the resonant frequency approached a steady state.

Based on the coagulation mechanism, the blood PT is determined as the end time of fibrin polymerization after the activation of thromboplastin and calcium [49]. As a method for coagulation analysis, the significant end-point from the FBAR frequency curve has to be picked for the determination of PT [11]. For this purpose, the first derivative of frequency-time curve was obtained in Figure 8b. The PT was given by df/dt = 0 and d^2^f/dt^2^ < 0, which is 10–60 s for different diluted blood samples. In addition, the curve profiles of the frequency-time response were closely dependent on the dilution factor of the blood sample, which could be attributed to the different rate of enzymatic cascade reaction and the degree of clotting. As expected, higher blood dilutions led to longer times and lower clot density because of a less compact fibrin network.

The coagulation process was also monitored by the FBAR devices coated with different polymers, including poly(methyl methacrylate), poly(ethylene terephthalate) and poly(dimethylsiloxane). The spin-coating processes and the changes of resonant performances were summarized in Table S1. For all the polymer coatings, the discrepancies of frequency shift and Q degradation due to mass loading were within 10%. The comparison of real-time frequency responses and PT values were provided in Supplementary Figure S5. It was discovered that the polyethylene-coated FBAR exhibited the best consistency with a standard coagulometer. Moreover, Müller et al. [11] reported that the PT values measured from QCM were about 15 s for the samples with the 1:2 blood dilution and 1:2 thromborel dilution. In comparison, the results of our samples with more activity (same blood dilution and no thromborel dilution) were about 30 s (Figure 8c). In addition, the tested PT values here are a little faster than those of cantilever-based microfluidic sensors [50] (made from poly(methyl methacrylate) and nickel) for the similar handling process. A possible reason is the procoagulant activity of polyethylene on the FBAR surface.

### 3.5. Comparison of the FBAR Sensor and a Standard Coagulometer

Usually, the analytical methods and the sample treatment procedure have an obvious influence on the blood coagulation kinetics. The coagulation times measured by QCM [11,51] devices and TEG [44] varied over a wide range. In this experiment, the blood samples were pretreated based on the recommendation of the coagulometer vendor and assayed using both the polyethylene-coated FBAR and the commercial coagulometer. The comparison between the results from the two methods could be accepted to demonstrate the accuracy of the measurement. Moreover, the obtained PT values of the samples without dilution (standard samples) were around 10 s, which is within the usual reference range for most clinical applications [52,53].

**Figure 9:**
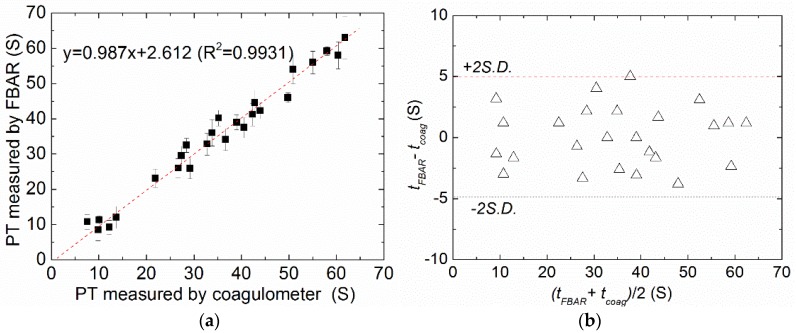
(**a**) Correlation between the PTs determined by the polyethylene-coated FBAR (*t*_FBAR_) and the results acquired by a commercial mechanical coagulometer (*t*_coag_); (**b**) The Bland–Altman plot of the measured data. The error bar is the standard deviation of six times measurements for each sample.

Figure 9a shows the correlation between the PTs determined by FBAR biosensor and the results acquired by the commercial mechanical coagulometer. It is found that the correlation (*R*^2^) between the two methods was 0.987. In this case, *R*^2^ = 0 would indicate that the FBAR biosensor is fully inaccurate while *R*^2^ = 1 would represent an exactly similar measurement. Furthermore, the Bland–Altman plot, which is a well-established procedure for the comparison of two different measurement techniques in science and clinical research, was used to evaluate the accuracy of the FBAR biosensor as shown in Figure 9b. In this special scatter diagram, the differences of the two methods were plotted against the mean value to estimate the fluctuation range of the accordance. For the results acquired by the FBAR biosensor and the commercial coagulometer, almost all data points lay in the given range of the mean plus/minus two times the standard deviation (±2SD), which suggests that about 95% of the values are within two standard deviations. In addition, the data showed that neither of the methods delivers systematically higher or lower values, suggesting a good consistency and clinical comparability between the FBAR biosensor and the commercial coagulometric methods.

### 3.6. Application for Warfarin Therapy

**Figure 10:**
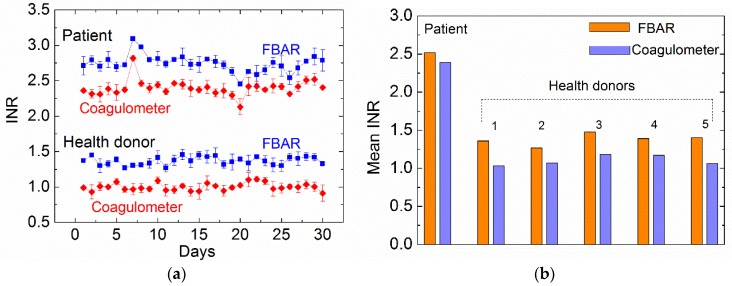
(**a**) International normalized ratio (INR) values of the patient and a healthy donor measured by the polyethylene-coated FBAR and the commercial coagulometer. The error bar is the standard deviation of six times measurements for the same sample. (**b**) Comparison of monthly mean INR between the two methods for the five honors.

To demonstrate the applicability of the FBAR biosensor, we performed the measurements of hemostatic parameters from a patient taking warfarin over a period of one month. According to clinical guidance, INR of the patient is the primary clinical indication for the therapy with warfarin or related oral anticoagulants. The safety and effectiveness of warfarin depends on keeping the INR within the target range, which is usually 2.0–3.0 [2,36]. Figure 10 shows the INR values of the patient and a healthy donor measured by different methods. All the blood samples were collected only once a day and each of the samples was tested six times by FBAR biosensor and the commercial coagulometer, respectively. All the data from the healthy persons showed similar profiles (provided in Supplementary Figure S6). For the same sample, the SD values were within a reasonable range. In fact, the SD values of the FBAR results showed a slight fluctuation, suggesting that the consistency of the fabrication process and experimental condition may be further improved for the clinic application.

Taking warfarin clearly resulted in an increased INR as seen in blood samples from the patient. The trends of coagulation results observed each day were similar to the two methods, although the FBAR biosensor measured a slightly higher INR than that of the commercial coagulometer. The anomalous changes in INR on day 7 (increase) and day 20 (decrease) were detected by both methods and indicate a higher risk of bleeding and thrombus, respectively. Figure 10b shows the monthly mean INR measured by FBAR biosensor and the coagulometer for the six honors. The small difference (~0.3) between the results measured by the two methods may be attributed to the fact that the ISI used in the calculation of INR was provided based on the commercial coagulometer but not calibrated specifically for the FBAR biosensors.

## 4. Conclusions

In summary, a high-performance shear mode FBAR biosensor based on an inclined *c*-axis AlN film was fabricated for monitoring the coagulation process of human blood. The wurtzite AlN film with the *c*-axis inclination angle of 22° was prepared to obtain an electric field component perpendicular to the normal direction. The resonances were excited at near 1.87 GHz and 3.15 GHz, corresponding to shear and longitudinal resonant modes, respectively. The shear mode resonance exhibited linear frequency downshifts with the increase of liquid viscosity, which is applied to monitor the sequential stages of blood coagulation, including cascade enzyme reaction, fibrin formation and fiber polymerization. The resonant frequency of the FBAR decreased along with a step-ladder time-dependent curve during blood coagulation. From both the control experimental studies and a real case measurement for a patient with warfarin treatment, we found that the clinically significant factor (PT) determined from the time-frequency curve of FBAR showed good consistency and comparability with the commercial coagulometer. One of the major advantages of this device is the micro-electromechanical fabrication, which allows a miniaturized device size and an ultra-small sample volume. Therefore, the proposed FBAR biosensors offer high potential for developing POC systems for hemostatic status monitoring. In further work, the methods of sample collection, storage, handling and data processing will be optimized for clinical applications.

## Figures and Tables

**Table 1:** Comparison between the FBARs with different structures.

Electrode Geometry	Crystal Orientation	Field Intensity (V/m) *	Electromechanical Coupling	Mean Allan Deviation
**Coplanar** **(lateral excited)**	**Normal *c*-axis**	1.73	0.32%	5.39 × 10^−8^
**Sandwiched** **(thickness excited)**	**Inclined *c*-axis**	6.67	0.68%	1.61 × 10^−8^

* The applied voltage between the electrodes was 10 V for the calculation.

**Table 2:** The resonant frequencies and Q factors of the FBAR devices measured in air.

	Resonant Frequency (MHz)		Q Factor	
	Bare	Polyethylene-Coated	Bare	Polyethylene-Coated
**Shear mode**	1876.4	1868.3	276	263
**Longitudinal mode**	3163.3	3150.4	169	176

**Table 3:** The frequencies and Q factors of shear and longitudinal resonances of polyethylene-coated FBAR measured in air, pure water and human blood.

	Resonant Frequency (MHz)			Q Factor		
	Air	Water	Blood	Air	Water	Blood
**Shear mode**	1868.3	1865.4	1863.1	263	203	129
**Longitudinal mode**	3150.4	3134.2	--	176	21	--

## Supplementary Materials

The following are available online at http://www.mdpi.com/2072-666X/9/10/501/s1, Figure S1: Structures of the film bulk acoustic resonator (FBAR) devices with sandwiched and coplanar electrodes. (a) Sandwiched electrodes; (b) Coplanar electrodes (lateral excited) with the gap of 5 μm; Figure S2: Calculated intensity and direction of electric fields for the FBAR devices with sandwiched and coplanar electrodes. (a) Sandwiched electrodes; (b) Coplanar electrodes (lateral excited) with the gap of 5 μm; Figure S3: Admittance curves of the FBAR devices with sandwiched and coplanar electrodes (lateral excited); Figure S4: Allan deviations of the FBAR devices with sandwiched and coplanar electrodes (lateral excited); Figure S5: The comparison of coagulation process monitored by the FBAR devices coated with polyethylene (PE), poly(methyl methacrylate) (PMMA), poly(ethylene terephthalate) (PET), poly(dimethylsiloxane) (PDMS) and Au (bare device). (a) Real-time frequency responses; (b) Prothrombin time (PT) values. The same blood samples with a dilution of 1:2 were tested for the devices. The polymers were spin-coated by the same process parameters; Figure S6 International normalized ratio (INR) values of the 5 healthy donors measured by the polyethylene-coated FBAR and commercial coagulometer. Table S1: The spin-coating process and the changes of resonant performances for different polymer layers on FBAR surface.
