# Supplementary material for: Shear Mode Bulk Acoustic Resonator Based on Inclined c-Axis AlN Film for Monitoring of Human Hemostatic Parameters
Source: Micromachines (Basel). 2018 Sep 30;9(10):501. doi: 10.3390/mi9100501 (PMC6215146; doi:10.3390/mi9100501)
Supplement: Supplementary file 1 [file micromachines-09-00501-s001.pdf]

micromachines

Supplementary Materials: Shear Mode Bulk Acoustic Resonator Based on Inclined *c*-Axis AlN Film for Monitoring of Human Hemostatic Parameters

Shuren Song [1], Da Chen [1,2,*], Hongfei Wang [1], Chaohui Li [1], Wei Wang [1], Wangli Yu [3], Yanyan Wang [4] and Qiuquan Guo [5,*]

1. The Comparisons between the TSM FBARs with Different Structures

1.1. The Exciting Electric Field

The intensity and direction of electric fields were calculated for the film bulk acoustic resonator (FBAR) devices with sandwiched and coplanar electrodes (Figure S1) using a finite element method. Both the devices are made by aluminum nitride (AlN) films and have membrane structure. As shown in Figure S2, the sandwiched electrodes produce a high intensity electric field with the large active area under the electrodes. In comparison, the lateral field only is excited in the small area between the coplanar electrodes (with the gap of 5 μm) and the intensity is about one-quarter of that produced by the sandwiched electrodes (Table 1 in the main body).

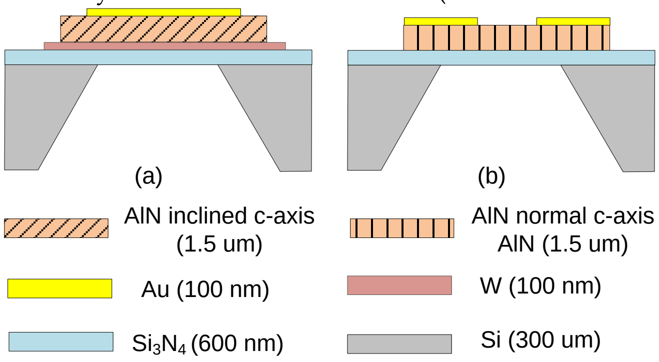

Figure S1. Structures of the film bulk acoustic resonator (FBAR) devices with sandwiched and coplanar electrodes. (**a**) Sandwiched electrodes; (**b**) Coplanar electrodes (lateral excited) with the gap of 5 μm.

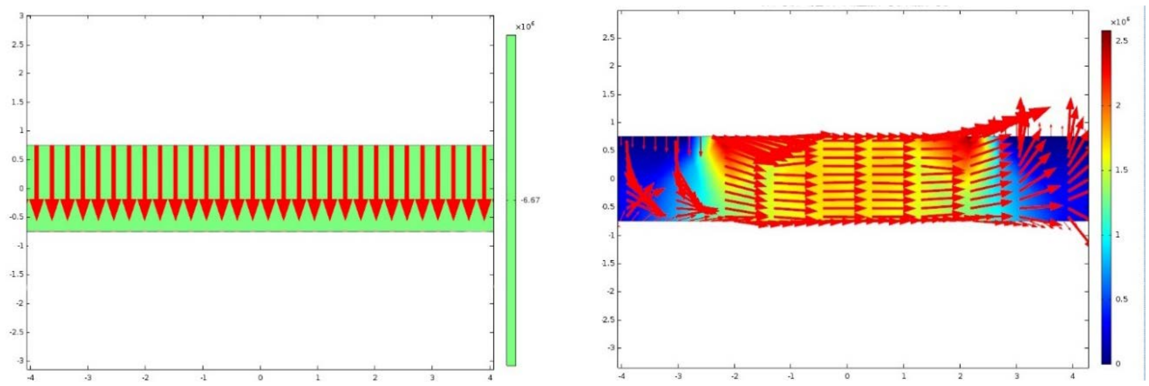

micromachines

(a) (b)

Figure S2. Calculated intensity and direction of electric fields for the FBAR devices with sandwiched and coplanar electrodes. (**a**) Sandwiched electrodes; (**b**) Coplanar electrodes (lateral excited) with the gap of 5 μm.

1.2 Electromechanical Coupling

Figure S3 shows the measured admittance curves of the FBAR devices with sandwiched and coplanar electrodes. The device effective electromechanical coupling content K2eff is related to the difference in series (fs) and parallel resonant frequencies (fp) in the admittance curve and can be evaluated using the following equation:

$$K_{eff}^2 = \frac{\pi^2}{4}\left(\frac{f_p - f_s}{f_p}\right)$$

As summarized in Table 1 (main body), the K2eff of sandwiched structure is more than twice that of lateral excited device.

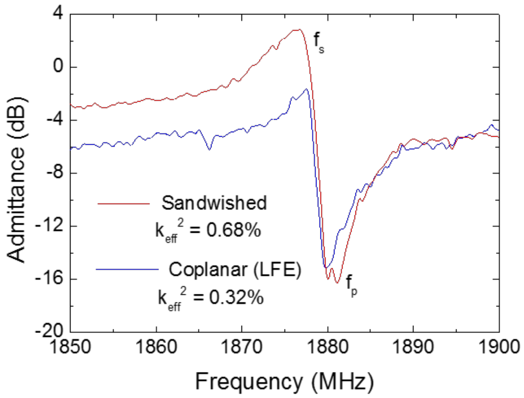

Figure S3. Admittance curves of the FBAR devices with sandwiched and coplanar electrodes (lateral excited).

1.3 Noise Level

The Allan deviation based upon the resonant frequency measurements is a standard parameter to evaluate the noise level in the time domain. Figure S4 shows the measured Allan deviation of the FBAR devices with sandwiched and coplanar electrodes at a sampling time of 25 s, using 100 samples. The mean Allan deviations are 5.39×10-8 and 1.61×10-8 for the devices with sandwiched and coplanar electrodes, respectively, suggesting the latter has a smaller noise level near the resonance.

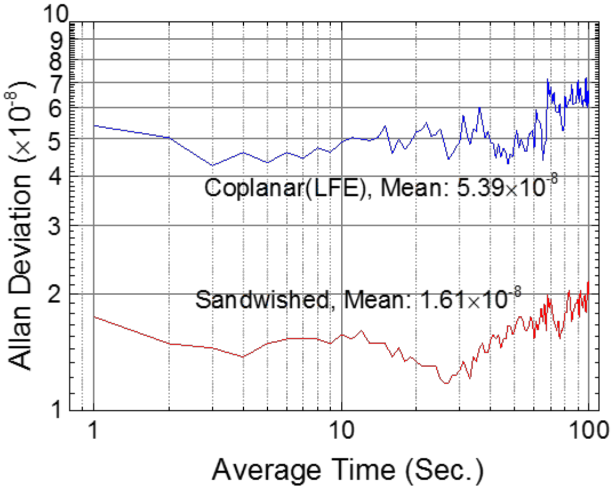

Figure S4. Allan deviations of the FBAR devices with sandwiched and coplanar electrodes (lateral excited).

2. The Real-Time Frequency Responses and PT Values of FBAR Devices Coated with Different Polymers

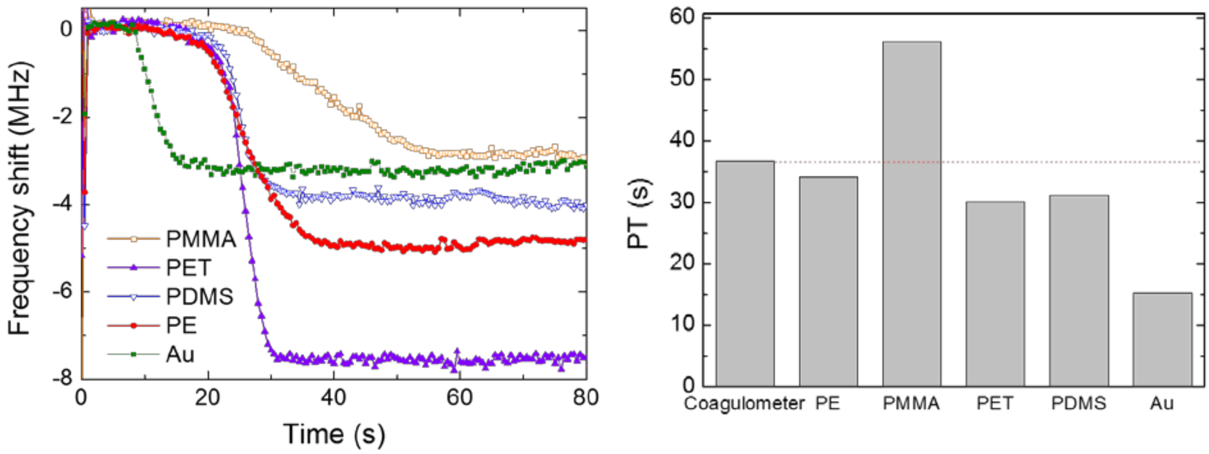

(a) **(b)**

Figure S5. The comparison of coagulation process monitored by the FBAR devices coated with polyethylene (PE), poly(methyl methacrylate) (PMMA), poly(ethylene terephthalate) (PET), poly(dimethylsiloxane) (PDMS) and Au (bare device). (**a**) Real-time frequency responses; (**b**) Prothrombin time (PT) values. The same blood samples with a dilution of 1:2 were tested for the devices. The polymers were spin-coated by the same process parameters.

Table S1. The spin-coating process and the changes of resonant performances for different polymer layers on FBAR surface.

	Solution	Rotation speed/time	Annealing temperature/time	Δf	ΔQ
**PE**	0.5% in decaline	6000rpm/40s	60 °C/20 min	8.1 MHz	13
**PMMA**	1% in toluene	4000rpm/40s	60 °C/20 min	7.6 MHz	No decrease
**PET**	0.3% in phenol	6000rpm/40s	60 °C/20 min	8.7 MHz	10
**PDMS**	0.5% in toluene	5000rpm/40s	60 °C/20 min	8.4 MHz	17

Δf : The frequency shift after coating

ΔQ : The degradation of Q factor after coating

3. The INR Values Measured by the FBAR Biosensor and the Commercial Coagulometer

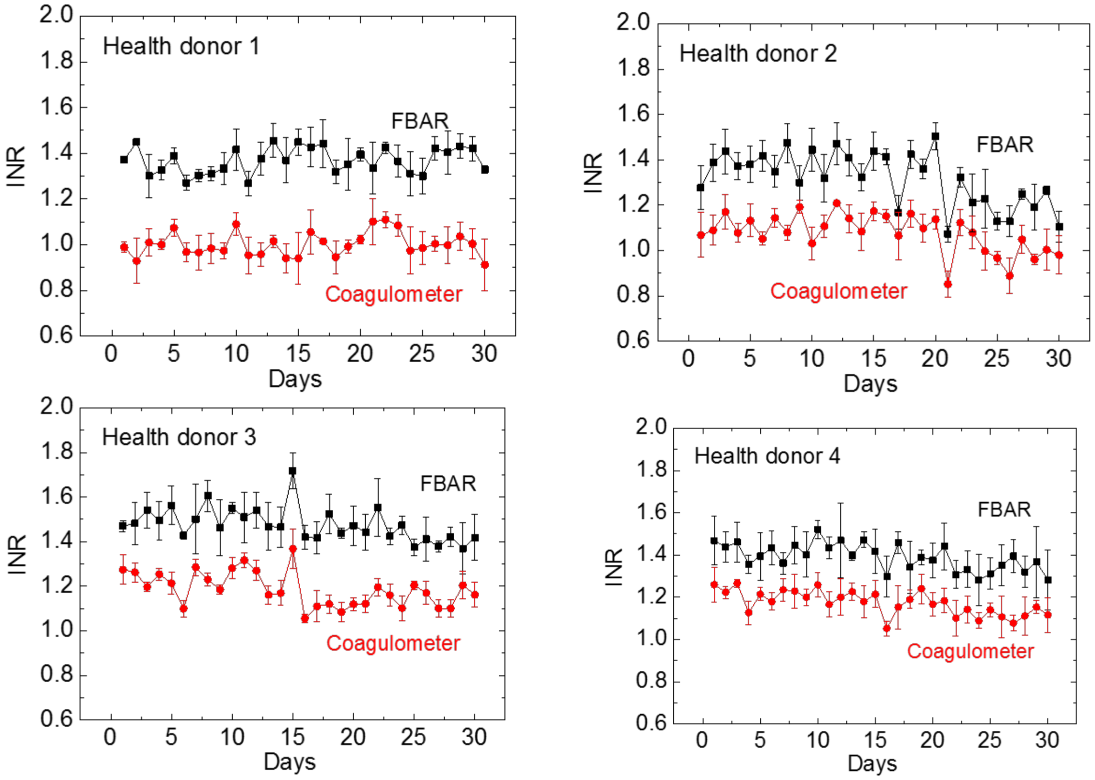

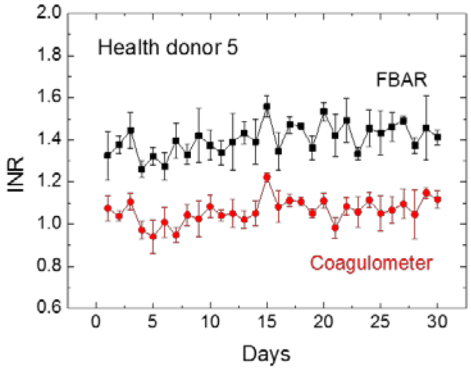

Figure S6. International normalized ratio (INR) values of the 5 healthy donors measured by the polyethylene-coated FBAR and commercial coagulometer.
